# Ultrasound-Guided Pulsed Radiofrequency in the Anterior Femoral Cutaneous Nerves to Treat Postherpetic Neuralgia in Anteromedial Thigh: A Case Report

**DOI:** 10.7759/cureus.93821

**Published:** 2025-10-04

**Authors:** Qing Yuan, Si Chen, Xulei Cui

**Affiliations:** 1 Department of Anesthesiology, Peking Union Medical College Hospital, Beijing, CHN

**Keywords:** anterior femoral cutaneous nerves, chronic pain management, pain, postherpetic neuralgia, pulsed radiofrequency

## Abstract

Postherpetic neuralgia (PHN) is a typical neuropathic pain after the eruption of the herpes zoster (HZ) rash. Pulsed radiofrequency (PRF) represents a minimally invasive, target-selective neuromodulatory intervention for PHN, with the dorsal root ganglion (DRG) serving as its primary therapeutic target. However, there is a paucity of evidence about the application of PRF to target peripheral nerves to treat PHN, which is easier to access with a lower risk of complications. We describe the successful management of a case by using PRF in the anterior femoral cutaneous nerves (AFCNs) to treat PHN in the anteromedial thigh in a 70-year-old male. This report highlights that PRF directed at peripheral nerves could also yield significant pain relief in chronic localized refractory PHN. Targeting peripheral nerves with PRF emerges as a viable treatment strategy for this patient population.

## Introduction

Postherpetic neuralgia (PHN) is characterized as neuropathic pain persisting for at least one month following the initial eruption of herpes zoster (HZ) rash. It is the most prevalent complication of HZ, estimated to affect 10-20% of all HZ patients [1]. Its incidence is escalating sharply with advancing age, causing persistent, often refractory neuropathic pain that severely impairs daily activities and overall quality of life [2]. This chronic condition arises from damage to sensory neurons within the dorsal root ganglion (DRG) and peripheral nerves, which is a typical but distinct neuropathic pain syndrome [3]. The standard pharmacologic management for PHN includes antiepileptics (gabapentin and pregabalin), antidepressants, topical agents, and other analgesics. But the application of analgesics has certain drawbacks, such as incomplete efficacy and systemic side effects [4]. Over the clinical course of PHN, the affected area may gradually diminish and become localized. Nevertheless, the residual focal neuropathic pain frequently remains persistent, unrelenting, and refractory to conventional therapeutic interventions.

In addition to oral analgesics and topical agents, there are some available interventional treatments for PHN, such as epidural injections, pulsed radiofrequency (PRF), nerve blocks, and spinal cord stimulation. However, a consensus is lacking regarding the optimal interventional therapeutic strategy for PHN. Among these interventional treatment options, PRF therapy shows a relatively promising long-term analgesic effect in subacute and chronic PHN [5]. PRF therapy is a minimally invasive procedure that delivers short bursts of high-voltage electrical currents adjacent to neural structures [6]. Unlike conventional continuous radiofrequency ablation, PRF generates a rapidly oscillating electromagnetic field without producing significant thermal tissue destruction. This unique physical interaction induces complex neuromodulatory effects at the cellular level, including alterations in synaptic transmission, suppression of pathologic neuronal firing, and modulation of pain-signaling pathways through both transient suppression of excitability and longer-term changes in gene expression [7]. 

In cases of PHN involving the trunk, PRF therapy is mostly directed at the DRG. PRF targeting peripheral nerves is mostly used in the treatment of PHN in the head and facial area [8], but seldom in the trunk. We report the successful management of a case by using ultrasound-guided PRF in the anterior femoral cutaneous nerves (AFCNs) to treat PHN in the anteromedial thigh in a 70-year-old male, since his pain was localized in the area innervated by the peripheral nerves.

## Case presentation

A 70-year-old male with a past medical history of herpes zoster, well-controlled hypertension, and type 2 diabetes for nine years presented to the pain clinic of a tertiary hospital. He had developed PHN in the dermatome of L3-L4 of the right leg, which had gradually become confined to the anteromedial thigh. The patient reported unrelieved pain despite medications including pregabalin and mecobalamin (500 μg, three times daily). The dosage of pregabalin had been initially 75 mg orally twice daily for the first week, which had then been titrated up to 150 mg twice daily. This pharmacological regimen had been maintained for over one year with limited efficacy. The pain was reported to be 5-6/10 according to the visual analog scale (VAS) with a constant, electric, shock-like sensation.

The patient presented to the clinic seeking interventional treatment for the residual pain in the anteromedial thigh. He also wished to reduce reliance on oral medication for symptom control. He had previously received PRF of the femoral nerve at another hospital two months ago, but the VAS didn’t improve after the treatment. A detailed sensory examination of the right lower limb showed allodynia and hyperalgesia on the anteromedial thigh. The skin sensation of the anteromedial thigh is innervated by the intermediate (IFCN) and the medial femoral cutaneous nerves (MFCN), collectively known as AFCNs [9]. We considered these two nerves to be suitable targets for PRF in this patient with refractory PHN affecting the anteromedial thigh. The patient provided written consent for the treatment and open access publication of the case and the ultrasound image.

**Figure 1:**
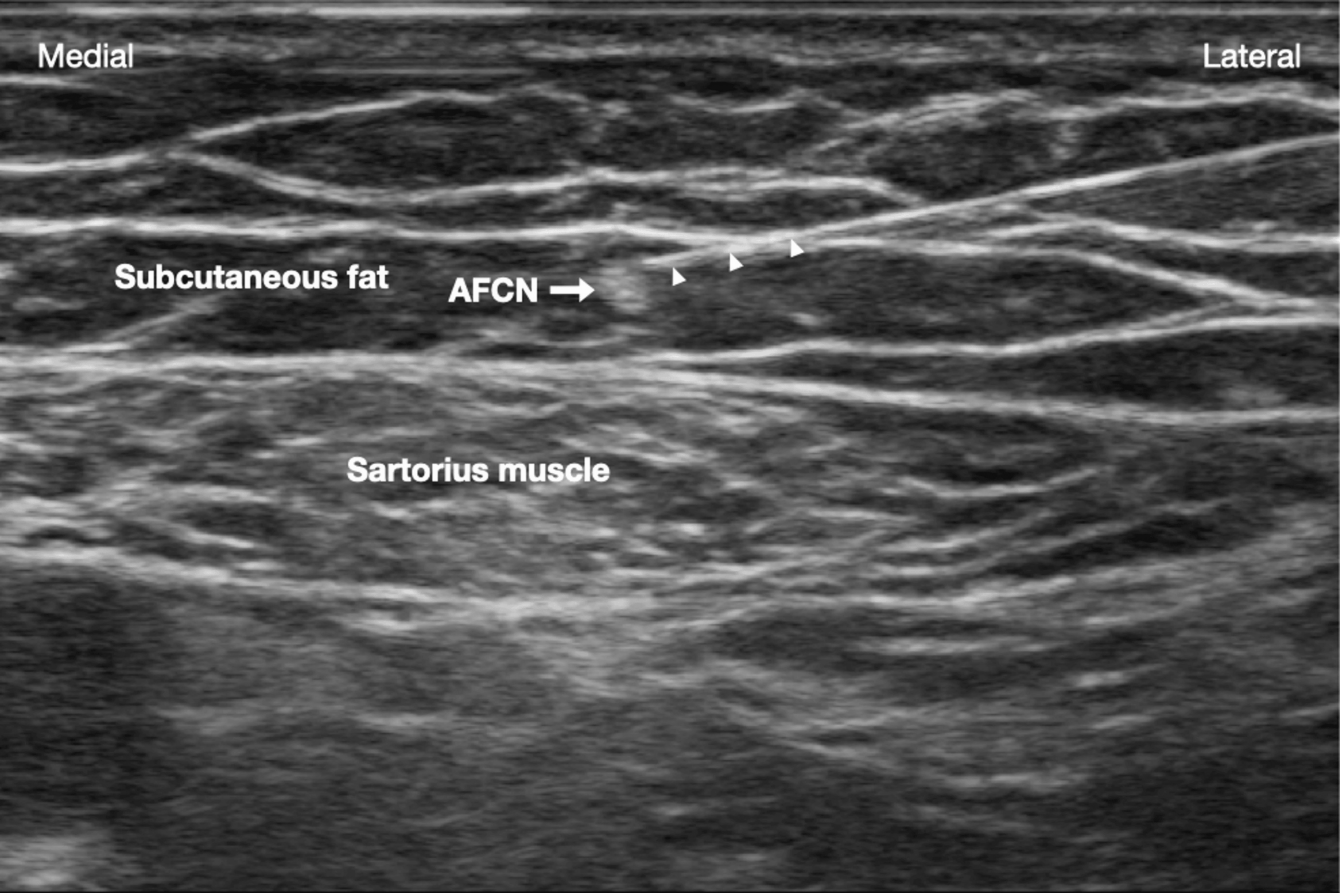
Pulsed radiofrequency of anterior femoral cutaneous nerves A linear probe (6-15 MHz) was placed transversely at the middle third of the anterior thigh to locate the sartorius muscle. Anterior femoral cutaneous nerves (AFCNs) are round hyperechoic structures found in subcutaneous fat overlying the sartorius, vastus medialis, and rectus femoris muscles. The needle was inserted from lateral to medial. White arrows: radiofrequency needle

Various methods have been reported for locating the IFCN and MFCN [10,11]. Here, we adopted the technique described by Pivec et al. [10] to identify these nerves. A linear probe (6-15 MHz) was placed at the middle third of the anterior thigh to locate the sartorius muscle. A careful scan was conducted to identify round hyperechoic structures in the subcutaneous fat overlying the sartorius, vastus medialis, and rectus femoris muscles. If the hyperechoic structures didn’t disappear when traced distally and proximally, we confirmed that they were branches of the IFCN and MFCN (Figure 1).

Sensory test stimulation (2 Hz, up to 0.5 V) was performed to verify the proper location of the needle tip. Then, PRF treatment was administered in the right IFCN and MFCN with a maximum temperature of 42 ℃ for 10 minutes (30 ms, 7 Hz, 80 V). The procedure was well-tolerated by the patient. No complications or signs of hematoma were observed. He was subsequently discharged in stable condition. The patient reported significant pain relief, with a reduction of more than 75% in VAS one week after the procedure and nearly complete pain relief one month postoperatively (Table 1). The patient was also able to stop the oral medication of pregabalin from 150 mg twice daily. No long-term complications, including parethesia, injury of surrounding tissue, or infection at the puncture site, were observed during follow-up.

**Table 1:** The change in the patient's visual analog scale score and medication PRF: pulsed radiofrequency

Time point	Visual analog scale score	Medication
Preop	6	Pregabalin 150 mg q12h; mecobalamin 500 μg tid
One day after PRF	3	Pregabalin 150 mg q12h; mecobalamin 500 μg tid
One week after PRF	1-2	Pregabalin 75 mg q12h; mecobalamin 500 μg tid
One month after PRF	1	None
Three months after PRF	0	None

## Discussion

PHN is a chronic neuropathic pain condition resulting from varicella-zoster virus (VZV) reactivation. PHN predominantly affects older adults, with prevalence significantly increasing with age, severely impairing quality of life [12]. The pathogenesis is complex, involving VZV infection of DRG or cranial nerve ganglia. When cellular immunity declines with aging or immunosuppression, latent VZV reactivates within DRG neurons. This directly causes ganglion inflammation and necrosis, leading to hypersensitivity and allodynia. Infiltration of inflammatory cells is also observed within the DRG, which would release inflammatory mediators contributing to both pain signaling and central sensitization [13].

Given the inadequacy of conservative pharmacotherapy (e.g., pregabalin, gabapentin, tricyclic antidepressants, lidocaine patches) for some patients with PHN, interventional procedures represent a crucial therapeutic option for persistent pain. These minimally invasive procedures encompass techniques such as nerve blockade, selective nerve destruction, intrathecal drug administration, PRF, and spinal cord stimulation. These therapies directly target lesion tissue through physical, chemical, or mechanical intervention. Among these interventional treatments, PRF demonstrates the most favorable long-term pain relief outcome [5].

PRF represents a minimally invasive, target-selective neuromodulatory intervention for PHN, with the DRG serving as its primary therapeutic target. Because it is believed that DRG plays a key role in inducing and maintaining PHN. Following varicella-zoster virus reactivation, persistent inflammatory changes and neuronal hyperexcitability within the DRG contribute substantially to both the initiation and maintenance of chronic neuropathic pain. PRF delivers controlled electromagnetic pulses to the affected DRG, inducing reversible electrophysiological alterations without thermal destruction, thereby addressing the core mechanisms of PHN pathogenesis originating in the DRG while preserving neural integrity.

Clinical studies have also reported therapeutic advantages of PRF over intercostal nerve treatments [14]. However, the virus also spreads along peripheral nerves to the skin, leading to sustained nerve hyperexcitability and spontaneous firing, causing PHN. The treatment of PRF targeting peripheral nerves has been used in PHN involving the facial and cranial area because the trigeminal ganglion is located deep in the middle cranial fossa. Many studies have demonstrated that this application of PRF is not only an effective and safe intervention but also provides satisfying analgesia in the orofacial PHN [8]. On the contrary, there is a paucity of evidence in the application of PRF targeting peripheral nerves to treat the PHN in the trunk.

Our patient's pain was localized to the anteromedial thigh, which is primarily innervated by AFCNs. This suggests that the ectopic spontaneous discharge originating from AFCNs contributed to his symptoms. Hence, we opted for a treatment strategy involving a strategy of PRF targeting the peripheral nerve of AFCNs instead of the DRG. The effect of the treatment turned out to be very satisfying. The mechanism of PRF in peripheral nerves involves its ability to reversibly inhibit nerve impulse transmission, particularly in small-diameter or unmyelinated C and Aδ fibers [15]. 

To the best of our knowledge, this is the first reported case in the medical literature of acute herpes zoster of the anterior thigh successfully treated with pulsed PRF therapy. The positive outcome in our case supports the theory that PRF neuromodulates peripheral sensory nerves, offering a potential treatment for refractory truncal PHN. Therefore, clinicians could consider targeting peripheral nerves as an alternative to the DRG. Peripheral nerves are more superficial and easier to access, whereas the DRG is situated deep within the intervertebral foramen. Reaching the DRG necessitates X-ray or CT guidance and is associated with greater risks, including pneumothorax, epidural hematoma, and intrathecal puncture.

This study has several limitations that should be acknowledged. First, as a report involving a single patient, our findings lack generalizability, and the outcomes may not be applicable to a broader population. Second, the absence of a control group makes it impossible to definitively establish a causal relationship between the intervention and the observed clinical improvement. Finally, the limited duration of our follow-up prevents us from assessing the long-term efficacy and potential late adverse effects of the PRF treatment. Future studies with larger sample sizes, controlled designs, and extended follow-up periods are necessary to validate our preliminary findings.

## Conclusions

We described the successful management of a case using PRF in AFCNs to treat PHN in the anterolateral thigh in a 70-year-old male. Our findings show that PRF directed at peripheral nerves could also yield significant pain relief in chronic localized refractory PHN. Hence, we hypothesize that PRF of peripheral nerves may be a promising treatment for truncal PHN. However, further research involving prospective and controlled clinical studies in well-defined patient populations is essential to validate the efficacy and long-term effects of PRF in targeting peripheral nerves.

## References

[1] Thompson RR, Kong CL, Porco TC, Kim E, Ebert CD, Acharya NR (2021). Herpes zoster and postherpetic neuralgia: changing incidence rates from 1994 to 2018 in the United States. Clin Infect Dis.

[2] Forbes HJ, Thomas SL, Smeeth L, Clayton T, Farmer R, Bhaskaran K, Langan SM (2016). A systematic review and meta-analysis of risk factors for postherpetic neuralgia. Pain.

[3] Adriaansen EJ, Jacobs JG, Vernooij LM, van Wijck AJ, Cohen SP, Huygen FJ, Rijsdijk M (2024). Herpes zoster and post herpetic neuralgia. Pain Pract.

[4] Patil A, Goldust M, Wollina U (2022). Herpes zoster: a review of clinical manifestations and management. Viruses.

[5] Wen B, Wang Y, Zhang C, Xu W, Fu Z (2020). Efficacy of different interventions for the treatment of postherpetic neuralgia: a Bayesian network meta-analysis. J Int Med Res.

[6] Sluijter ME, Imani F (2013). Evolution and mode of action of pulsed radiofrequency. Anesth Pain Med.

[7] Sam J, Catapano M, Sahni S, Ma F, Abd-Elsayed A, Visnjevac O (2021). Pulsed radiofrequency in interventional pain management: cellular and molecular mechanisms of action - an update and review. Pain Physician.

[8] Wang C, Dou Z, Yan M, Wang B (2023). Efficacy and safety of pulsed radiofrequency in herpes zoster related trigeminal neuralgia: a systematic review and meta-analysis. J Pain Res.

[9] Riegler G, Pivec C, Jengojan S, Mayer JA, Schellen C, Trattnig S, Bodner G (2021). Cutaneous nerve fields of the anteromedial lower limb-determination with selective ultrasound-guided nerve blockade. Clin Anat.

[10] Pivec C, Bodner G, Mayer JA (2018). Novel demonstration of the anterior femoral cutaneous nerves using ultrasound. Ultraschall Med.

[11] Gong WY, Li CG, Fan K (2022). A novel ultrasound-guided technique for intermediate femoral cutaneous nerve block. Minerva Anestesiol.

[12] Yawn BP, Saddier P, Wollan PC, St Sauver JL, Kurland MJ, Sy LS (2007). A population-based study of the incidence and complication rates of herpes zoster before zoster vaccine introduction. Mayo Clin Proc.

[13] Garry EM, Delaney A, Anderson HA (2005). Varicella zoster virus induces neuropathic changes in rat dorsal root ganglia and behavioral reflex sensitisation that is attenuated by gabapentin or sodium channel blocking drugs. Pain.

[14] Tang J, Zhang Y, Liu C, Zeng A, Song L (2023). Therapeutic strategies for postherpetic neuralgia: mechanisms, treatments, and perspectives. Curr Pain Headache Rep.

[15] Tun K, Cemil B, Gurcay AG (2009). Ultrastructural evaluation of pulsed radiofrequency and conventional radiofrequency lesions in rat sciatic nerve. Surg Neurol.

